# The Role of NKG2D in Vitiligo

**DOI:** 10.3389/fimmu.2021.624131

**Published:** 2021-02-26

**Authors:** Lourdes Plaza-Rojas, José A. Guevara-Patiño

**Affiliations:** ^1^Department of Cancer Biology, Loyola University Chicago, Chicago, IL, United States; ^2^Department of Surgery, Loyola University Chicago, Chicago, IL, United States

**Keywords:** vitiligo, NKG2D, T cells, horror autotoxicus, Hsp70, oxidative stress

## Abstract

Vitiligo is an acquired multifactorial disease that affects melanocytes and results in skin depigmentation. In this review, we examine the role of cells stress and self-reactive T cells responses. Given the canonical and non-canonical functions of NKG2D, such as authenticating stressed target and enhance TCR signaling, we examine how melanocyte stress leads to the expression of ligands that are recognized by the activating receptor NKG2D, and how its signaling results in the turning of T cells against self (melanocyte suicide by proxy). We also discuss how this initiation phase is followed by T cell perpetuation, as NKG2D signaling results in self-sustained long-lasting T cells, with improved cytolytic properties.

## Introduction

At the end of the eighteenth century, Paul Ehrlich introduced the concept of horror autotoxicus, which describes the body's innate aversion to immunological self-destruction. This model remains valid, as the immune system is entrusted with protecting the host against infections while avoiding autoimmunity. It is well-accepted that the killing of target cells by T cells occurs through recognition of the peptide-MHC Class I complex via the T cell receptor (TCR). In the thymus, T cells undergo positive and negative selection based on the TCR signal strength (1). T cells that recognize self-antigens with strong affinity undergo negative selection. In contrast, T cells that weakly respond to self-antigens undergo positive selection and populate the periphery as mature T cells. Thus, while self-reactive T cells are present in the periphery, their TCR interactions with peptide-MHC Class I complex are insufficient to mediate activation. Moreover, additional peripheral mechanisms are also in place to prevent their activation, namely regulatory T cells (Tregs) (2, 3).

Under certain pathophysiological conditions, T cells are responsible for several autoimmune disorders, including vitiligo. Vitiligo is an autoimmune disease characterized by the progressive loss of skin pigmentation as a result of melanocyte destruction. In this context, T cells are known to target and kill melanocytes, the pigment-producing cells in the skin. Vitiligo is the most common skin pigmentation disorder, affecting 0.1–2% of the population worldwide (4, 5), with no sex bias (6–9). While it can affect people at all ages, vitiligo appears more frequently before 20 years of age and its early onset (during childhood) is associated with hereditary disease (5, 7, 9–11). Vitiligo is characterized by white lesions on the skin, and the associated psychological and social effects make patients more prone to depression and low self-esteem (12). Regrettably, there are currently no FDA-approved treatments for vitiligo (13). Although great progress has been made throughout the past decades in elucidating the mechanisms that cause this disease, it remains incurable.

While T cells turn against melanocytes for reasons that remain largely unknown, recent studies suggest that CD8 T cells kill melanocytes through the recognition of stressed/damaged cells via Natural Killer Group 2D (NKG2D). NKG2D is a type II transmembrane receptor encoded by the killer cell lectin-like receptor subfamily K member 1 (Klrk1) gene (14, 15) expressed by CD8 T cells, NK cells, NKT cells, and a subset of γδ T cells, and by CD4 T cells in some pathologies (16, 17). Although NKG2D is expressed in both NK and CD8 T cells, the mechanisms of action differ. In NK cells, NKG2D signaling is sufficient to mediate direct killing of target cells (18). However, in CD8 T cells, NKG2D requires the concurrent activation of the TCR. In this case, NKG2D enhances TCR activation and thus, T cell function (18–20).

A number of elements are thought to contribute to the initiation of vitiligo, including genetic predisposition (21, 22), reduced proliferative capacity of melanocytes, increased oxidative stress in melanocytes, the subsequent expression of “danger” signals, reduced presence and function of Tregs, and increased activation of a self-reactive T cell repertoire (7, 23, 24). Moreover, there is evidence of an association between early inflammatory events (e.g., exposure to UV light, a bleaching agent, phenols, or trauma) and vitiligo (25, 26). We believe this is of significance, as the expression of NKG2D ligands (NKG2DL) is upregulated in response to cell stress (insult), rendering these cells visible to NKG2D-expressing immune cells (14, 18–20, 27). The ligands for NKG2D are composed of a variety of stress molecules, including the major histocompatibility complex (MHC) class-I-related chain (MIC) A/B and ULBP binding proteins 1–6 in humans (16) and members of the Rae-1 and H60a-c families in mice (28, 29). Importantly, NKG2DL expression on the cell surface is rigidly controlled. In contrast to physiological conditions in which few or no NKG2DL are expressed, their expression is induced under stress conditions, such as infection and transformation (28, 29). Of significance, proinflammatory signals such as IFN-α (30) and TLR4 and TLR7/8 signaling (28), as well as the ataxia telangiectasia mutated and Rad3-related (ATM/ATR) DNA damage response pathway also result in surface expression of these ligands (20, 27, 31, 32). Thus, the canonical function of NKG2D is to authenticate the stressed/damaged feature of the target cells. This cell authentication occurs in part by favoring the TCR signaling.

Notably, studies from our group have established in animal models that NKG2D signaling also leads to the development of long-lasting CD8 T cells with enhanced cytolytic function (33–35). NKG2DL upregulation and NKG2D signaling activation in effector CD8 T cells play a role in the onset or development of some autoimmune diseases, including vitiligo, rheumatoid arthritis, celiac disease, type 1 diabetes, alopecia areata, systemic lupus erythematosus (SLE), among others (36–41). Moreover, inhibiting NKG2D engagement can prevent inflammation and disease development in some models of type 1 diabetes (42), vitíligo (30), and other inflammatory diseases (16).

Here, we describe the relationship between multiple factors that can lead to vitiligo development with a main focus on the involvement of CD8 T cells and NKG2D signaling.

## Melanin Synthesis, UV Exposure, and Oxidative Stress

While multiple factors may induce the development of vitiligo, a dysfunction of the redox balance has been largely observed both systemically and in active lesions of vitiligo patients, causing an excessive accumulation of reactive oxygen species (ROS) and inducing cellular stress (43–45). Oxidative stress can arise when molecular oxygen (O_2_) is converted into oxygen radicals and the production of these species surpasses the anti-oxidant capacity of the cell, causing oxidation of cellular structures and DNA and potentially leading to cell death (46–49). A number of pathologies are associated with excessive ROS generation, which can also lead to inflammation, particularly in autoimmune diseases (50). Given the effects of ROS on inflammation as well as the active role of immune cells in the development of vitiligo, a relationship between oxidative stress and the immune system is apparent in the onset of the disease.

Melanocytes are derived from the neural crest and their main function is to produce the pigment melanin, which is responsible for skin and eye color (51). Keratinocytes, the most abundant cell type in the epidermis, regulate melanocytic functions, including proliferation, melanogenesis, and differentiation (52). Melanocytes and keratinocytes are arranged in epidermal melanin units, in which 1 melanocyte is surrounded by 36 keratinocytes. While its synthesis occurs in melanocytes, melanin is transported to the surrounding keratinocytes (53). This pigment is generated from the oxidation of tyrosine in a multistep reaction during which free radicals may arise (54). These reactions are highly regulated and carried on by a multienzyme complex unique to melanocytes, including the enzymes tyrosinase, TYRP1 and TYRP2 (55).

Exposure to UV light and its absorption by melanocytes causes photo-oxidation of melanin, generating superoxide radicals (54), which in turn induce melanin biosynthesis (56). Melanin creates a supranuclear cap to protect DNA and prevent or reduce its damage (57) and it has also been shown to act as an antioxidant and a free radical scavenger (58–60). However, not only does melanin biosynthesis consist of several oxidation reactions, it also requires energy production via mitochondrial respiration (61). Even though ROS generation has an important role in cell signaling, increased or altered mitochondrial activity may lead to excessive ROS production (62) that results in detrimental effects and apoptosis, as is the case in vitiliginous melanocytes. Although oxidative stress has been observed in both melanocytes and keratinocytes, the latter are less likely to undergo apoptosis. Instead, keratinocytes become senescent in response to UV irradiation (63, 64), and exposure to ROS induces the production of inflammatory cytokines by keratinocytes (65, 66).

Despite an increased O_2_ consumption rate, mitochondrial respiration and energy production is impaired, while ROS production is increased in vitiliginous melanocytes (67). Higher levels of ROS peroxynitrite and H_2_O_2_ are found in the skin of vitiligo patients, in concert with lower concentration of antioxidants and reducing enzymes (43, 68–70). High concentrations of H_2_O_2_ disrupt melanin synthesis by inhibiting tyrosinase and dihydropteridine reductase (71). Calreticulin, an endoplasmic reticulum (ER) protein that regulates Ca^2+^ homeostasis and signaling, is also modulated by H_2_O_2_, which increases calreticulin expression and translocation to the cell surface of melanocytes. This is associated with higher melanocyte apoptosis and production of pro-inflammatory cytokines IL-6 and TNF-α in vitiligo (72). As will be further discussed, pro-inflammatory cytokines have an important role in the vitiligo-associated immune response.

High ROS can also induce ER oxidation; in particular, H_2_O_2_ can interfere with the ion channel TRPM2, resulting in higher Ca^2+^ influx into the cell and the mitochondria (73). Increased mitochondrial Ca^2+^ concentration ([Ca^2+^]), in addition to augmented ROS production, induces a reduction of the mitochondrial membrane potential (ΔΨm) in melanocytes and the circulating mononuclear cells of vitiligo patients (44, 72). While melanin is known to chelate Ca^2+^ and control intracellular [Ca^2+^], protecting the cell from DNA damage caused by ROS or UV rays, eventually, these alterations may lead to cytochrome c release and apoptosis (74, 75). In the ER, ROS induces accumulation of dysfunctional and unfolded proteins, triggering the unfolded protein response (UPR) within vitiliginous melanocytes, potentially leading to cell death (76). Moreover, aberrant self-proteins, perceived as foreign antigens, can elicit immune responses (77). Therefore, oxidative stress in vitiligo skin is likely to precede the autoimmune response. In particular, NKG2DL expression can be triggered by ROS in multiple cell types and in cancer (78–83). NKG2D-mediated activation of CD8 T cells may thus play a significant role in the development of vitiligo, as discussed below.

## NKG2D, Stress Signals, and T Cells

NKG2D is an activating receptor that is expressed by many cytotoxic lymphocytes and binds to a variety of ligands (i.e., MICA and Rae-1 in human and mouse, respectively) expressed on stressed cells. Studies including ours have shown that the function of these ligands is to enable stressed cell recognition and destruction in a suicide by proxy mechanism, facilitated by immune cells expressing NKG2D (34, 84, 85). Interestingly, increased expression of NKG2DL has been shown in response to multiple insults, including oxidative cell stress (86, 87). Moreover, studies have shown an association between CD8 T cells expressing NKG2D and various autoimmune diseases, including skin conditions such as alopecia areata (88–90), vitiligo, pancreatitis, diabetes (91), and celiac disease (39, 92). For example, in type 1 diabetes, NKG2D engagement is necessary for the development of the disease (42) and destruction of β-cells in the pancreas by CD8 T cells has been compared to melanocyte death in skin patches of vitiligo patients (93, 94).

In a mouse model of vitiligo, we show that engagement of NKG2D results in exacerbation of CD8 T cell-mediated vitiligo (34). Consistent with this, melanocyte-reactive T cells are further activated and drawn to stressed melanocytes that express NKG2DL (19). Moreover, NKG2D-expressing T cells are enriched in vitiligo patients (30). Thus, understanding the mechanisms that regulate NKG2DL expression on stressed melanocytes can lead to development of therapeutic approaches targeting the interactions of NKG2D^+^ self-reactive CD8 T cells with melanocytes in vitiligo.

The expression of NKG2DL is upregulated in response to cell stress, rendering stressed or altered cells visible to NKG2D-expressing CD8 T cells. Studies have shown that stress signals induce the expression of NKG2DL on most cell types, including melanocytes (30). Increased expression of such signals in healthy tissue may induce the destruction and inflammation associated with autoimmune diseases (36, 37, 39, 41, 42, 88, 95–97). In fact, melanocyte-reactive T cells, normally suppressed by Tregs, have been found in circulating blood of individuals that do not exhibit vitiligo (98). While such self-reactive cytolytic T cells can be exploited for anti-tumor therapy, loss of functional Treg cells can result in the onset of vitiligo (98, 99).

NKG2DL expression on melanocytes may also drive the onset of the disease. This was demonstrated in a mouse model which revealed that NKG2DL expression on target cells is sufficient to induce vitiligo (34). Accordingly, melanocyte-reactive T cells also have a higher NKG2D expression. In particular, a subset of tissue-resident memory CD8 T (T_RM_) cells populating lesion areas in the skin of vitiligo patients was shown to have increased NKG2D levels and to be responsible for the increased production of IFN-γ and TNF-α (30). This T_RM_ cell activation was mediated not only by IL-15, a key cytokine in memory formation, but also by skin dendritic cells (DCs) expressing human NKG2DL MICA/B (30). Engagement of NKG2D on CD8 T cells in combination with IL-15 stimulation can trigger the killing of target cells, a feature of autoimmune diseases (39, 84, 95, 100–102). While NKG2D plays an important role in the effector phase of T cells, we have shown that engagement of this receptor also has long-term effects, including the promotion of memory formation (35). In the mentioned study, we show that signaling through NKG2D mediates a process that we termed memory certification. We found that temporary blockade of NKG2D signaling during the effector phase resulted in the formation of highly defective memory CD8 T cells characterized by altered expression of the ribosomal protein S6 and epigenetic modifiers, suggesting modifications in the T cell translational machinery and epigenetic programming. Based on these data, we concluded that NKG2D signaling during this initial effector phase, poises NKG2D engaged cells with a certification or molecular accreditation that results in their optimal development as memory T cells. This process of certification guarantees that the memory compartment is populated with CD8 T cells that have demonstrated their ability to kill the correct targets through a two-step process that utilizes the TCR and NKG2D signaling. Interestingly, in this system, NKG2D-certtified memory T cells will be able to kill melanocytes independently of NKG2D, thus resulting in the destruction of healthy melanocytes. We postulate that melanocyte-reactive CD8 T cells that receive NKG2D signaling during the killing phase will also persist as memory cells and potentially enrich the skin TRM population in vitiligo patients.

Heat shock protein are stress-inducible chaperones that protect cells from undergoing apoptosis through several mechanisms, including the binding and renaturing or degradation of misfolded proteins during the stress response (103). In particular, Hsp70 expression in tumor cells has been shown to elicit both innate and adaptive immune responses (104–108). In vitiligo, stressed melanocytes increase the expression of Hsp70, which binds and transports potentially immunogenic antigens to the MHC complex, allowing for their presentation on the cell surface to cytotoxic T cells (109). Moreover, free Hsp70 also triggers an immune response by interacting with DC surface receptors (107, 110, 111), inducing the expression of human NKG2DL MICA (107). This acts as a danger signal, rendering stressed cells visible to both NK and CD8 T cells expressing NKG2D. Hsp70 expression can be induced not only by physical and chemical stress, but also in physiological conditions, such as under high ROS production, as seen in vitiligo and other pathologies (110, 112, 113). Expression of both inducible HSP70 and NKG2DL is controlled by the transcriptional activator heath shock factor 1 (HSF1) (83). In response to cell stress, HSF1 is released in the cytoplasm. Following nuclear translocation, HSF1 binds to the promoter regions of both HSP70i and NKG2DL, initiating their transcription. These observations raise a novel paradox: while the canonical function of intracellular HSP70i is cytoprotectant, expression of NKG2DL results in cell suicide by proxy. While it is not known how melanocytes survive or render themselves visible for killing after cell stress, we believe that focusing on answering this question may in turn help identify actionable therapies in vitiligo.

Environmental insults can trigger inflammation and result in the onset of vitiligo, especially in adulthood (25, 26). For example, monobenzone, a skin bleaching agent is used to complete skin depigmentation in vitiligo patients. Monobenzone induces ROS generation and oxidative stress in the skin (114), further contributing to the autoimmune response in vitiligo. In addition, monobenzone is converted into a reactive quinone by tyrosinase (114); these quinone products bind covalently to proteins in melanocytes, forming aberrant antigens targeted by T cells (115). Therefore, monobenzone induces a systemic T cell response, effectively targeting all melanocytes in the skin.

## The Inflammatory Microenvironment in Vitiligo

Cytokines have a crucial role in regulating immune responses. High levels of cytokines are often found in vitiligo patients, including cytokines corresponding to Th1, Th17, and innate cell responses (116). Interestingly, keratinocytes in vitiligo lesions aberrantly produce IL-1, IL-6, and TNF-α, which inhibit melanocyte function (65, 66) and elicit an inflammatory response.

The production of pro-inflammatory cytokines allows for enhanced T cell recruitment and activation, resulting in increased presence of activated CD8 T cells in vitiligo lesions (13, 117–122). Produced by multiple immune cell types, IFN-γ is associated with vitiligo. IFN-γ promotes the production of other cytokines and chemokines, and the recruitment of T cells as well as other types of immune cells (123). Studies show that IFN-γ directly induces melanocyte apoptosis (124, 125) and its signaling enhances CD8 T cell function and expansion (126). Notably, we and others have demonstrated that NKG2D signaling upregulates IFN-γ production (18, 33). IFN-γ also promotes the expression of MHC Class I and therefore antigen presentation for CD8 T cells. IFN-γ is also responsible for stimulating the production of CXCL9 and CXCL10 in keratinocytes, which engages the chemokine receptor CXCR3 on melanocytes and triggers apoptosis, further contributing to the disease (127).

TNF-α is also produced at high levels in vitiligo (128). While its role as an apoptotic mediator has been shown in multiple cell types (129), in vitiligo, TNF-α inhibits melanogenesis by activating the transcription factor NF-κB (130). In addition, it induces the expression of ICAM-1 on melanocytes (131), allowing for the attachment of T cells and triggering cytotoxicity (132). Moreover, TNF-α inhibits tyrosinase and Trp1 activity, both essential for melanin synthesis (61). ROS production is also increased by TNF-α, further promoting stress signals in vitiligo melanocytes.

While keratinocytes can contribute to the production of inflammatory factors, melanocyte-reactive CD8 T cells are sufficient to initiate the development of vitiligo, as it has been shown that perilesional T cells from vitiligo patients can recognize and kill melanocytes in healthy skin (122). Activated self-reactive T cells remain in the skin and differentiate into T_RM_ cells. This differentiation is mediated by IL-15 derived from keratinocytes (133). Blockade of IL-15Rβ (CD122) reduces IFN-γ production and can eliminate skin T_RM_ and reverse vitiligo (133).

Another indicator of an existing melanocyte-reactive T cell population is the development of vitiligo in melanoma patients who receive immunotherapy (134–140). In patients without the right stimuli, these T cells do not mount a response, but can potentially be activated for the treatment of cancer. This observation also indicates the possibility to treat melanoma with agents known to trigger vitiligo, such as skin bleaching agents (114, 115).

In addition to a high production of pro-inflammatory cytokines, there is a corresponding reduction in suppressive cytokines in the skin of vitiligo patients. Tregs maintain immune tolerance by controlling self-reactive cytotoxic T cells. In healthy adults, Tregs constitute about half of the CD4 T cell population in the skin (141). In vitiligo, however, a much lower percentage of Tregs is often found surrounding lesions (23), which has been associated with reduced levels of Treg growth and differentiation factors TGF-β and IL-10 (142, 143). In particular, low TGF-β concentrations in both serum and skin have been found to correlate with skin depigmentation in vitiligo (142, 144). In addition, there is an association between reduced expression of CCL22, a skin-homing chemokine, and Treg infiltration in vitiligo skin, suggesting impaired migration of Tregs to the skin in vitiligo patients (24). These events can thus result in the alteration of the Treg/cytotoxic T cell ratio, impaired Treg differentiation, and increased inflammation in vitiligo.

In summation, the imbalance of pro-inflammatory and suppressive cytokines further contributes to disease progression. The interplay of these messengers, the immune system, and the affected skin cells is being actively investigated to identify novel therapeutic avenues.

## Genetic and Transcriptional Alterations in Vitiligo

Genetic predisposition is heavily linked to an overreactive immune system and patients often exhibit more than one autoimmune disease. While the inflammation and stress observed in vitiligo patients can have a variety of causes, including environmental, there is also a high risk of vitiligo heritability, with about 8% of patients having at least one affected relative (7). Gene analysis studies have revealed a higher expression of Class II MHC haplotypes HLA-DR and HLA-DQ in Caucasian patients resulting from a polymorphism in a super-enhancer within the Class II region (6, 145). Moreover, there is a correlation in the expression of such MHC haplotypes and a higher production of pro-inflammatory cytokines IFN-γ and IL-1β (6). In addition, mutations affecting other loci implicated in immune regulation have been associated with vitiligo and other autoimmune diseases. These genes include those encoding IL-2Rα (146), the TCR signaling regulator UBASH3A (146), and CCL20 receptor CCR6 (147), among others (21). While these reports indicate that there is an association between immune regulation and the onset of autoimmunity and vitiligo, there is also evidence that some polymorphisms lead to reactivity specifically against melanocytes. These include peptides derived from tyrosinase, the transmembrane protein OCA2, and melanocortin 1 receptor (MC1R), all of which are naturally processed and presented as antigens by Class I MHC molecules (5, 148, 149). This, in combination with higher MHC expression, increases the potential for the presentation of melanocyte-specific antigens and for self-reactive T cells targeting these cells.

Reduced function and number of the tolerogenic Tregs has also been associated with the development of vitiligo. Genetic polymorphisms in Foxp3, a transcription factor critical for Treg differentiation, and transcription factor FoxP1 (147), also critical for Treg homeostasis (150, 151), have been associated with Treg dysfunction in vitiligo (23, 152–154). Similarly, variants in the genes encoding TGF-β receptor II (155) and IL-10 (156) may affect Treg-mediated immune suppression in vitiligo. As mentioned, both TGF-β and IL-10 are cytokines essential for Treg differentiation and development (157–159).

Additionally, transcriptional analyses have demonstrated altered expression of factors involved in proliferation, apoptosis and regeneration of melanocytes (160). This is supported by the observation that vitiliginous melanocytes display poor proliferative capacity and cannot be sub-cultured *in vitro* (161, 162).

Aberrant expression of miRNAs has also been observed in vitiligo (163–165). In particular, miRNA-211 is highly expressed in healthy melanocytes, but its expression is severely reduced in non-pigmented melanoma and in the vitiligo cell line PIG3V (22). miRNA-211 target genes have functions involved in cell respiration and metabolism, including mitochondrial complexes I, II, and IV. In consequence, reduced miRNA-211 expression in vitiligo is associated with increased O_2_ consumption and may partially explain the oxidative stress observed in patients (22, 166).

## Conclusions

**Figure 1:**
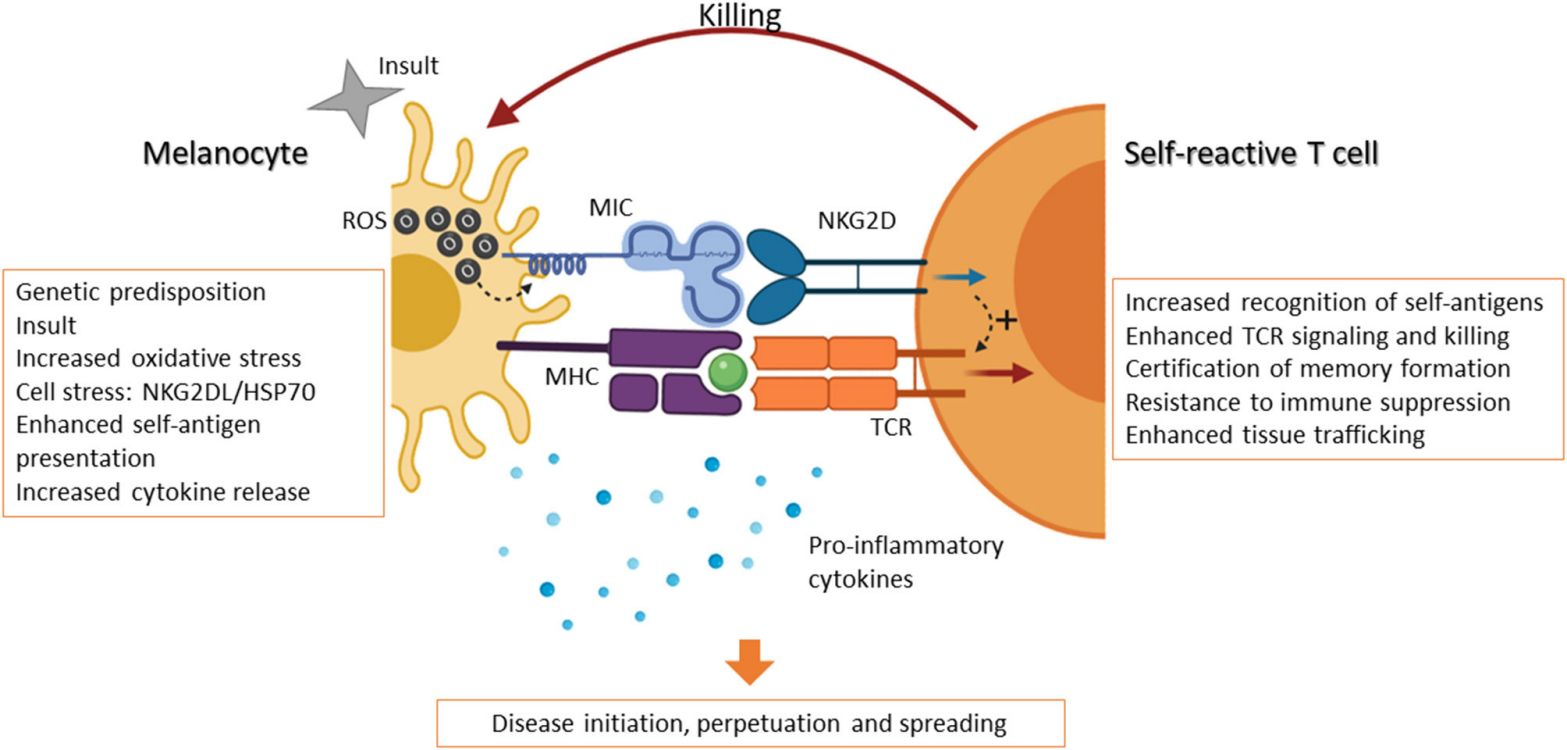
NKG2D-expressing T cells initiate and perpetuate vitiligo. The occurrence of vitiligo can be associated with genetic predisposition and hereditary disease, which in turned is linked to an overreactive immune system. Expression of certain MHC variants, for example, can lead to increased self-antigen presentation. However, other contributing factors can trigger the onset of the disease. An inflammatory event, or insult, may lead to increased ROS production and oxidative stress in melanocytes and their surroundings. In response to this, the production of cytokines by immune cells and keratinocytes can further induce inflammation and an immune response. Melanocytes in turn express “danger” signals, including NKG2D ligands (e.g., MICA/B in humans) and chaperone proteins, such as hsp70. Self-reactive cytolytic T cells are thus able to recognize melanocytes through the TCR and become activated via the engagement of NKG2D, whose signaling further enhances the downstream effects of TCR signaling, including cytotoxicity. Moreover, NKG2D activation certifies these self-reactive T cells to become memory cells and renders them more resistant to immune suppression. These certified T cells have enhanced local trafficking and are able to continue killing melanocytes. These events, in combination with reduced suppression by dysfunctional Treg cells, result in the onset, perpetuation, and spreading of vitiligo. Created with BioRender.com.

Vitiligo is a complex disease with multiple causal factors, including genetic predisposition, enhanced self-antigen presentation, and increased pro-inflammatory signals. Inflammation is known to induce DNA and cell damage and increased ROS production, triggering the expression of NKG2DL on melanocytes. It is well-accepted that the interaction between the autoantigen:MHC-I complex and the self-reactive TCR on CD8 T cells is weak. However, under stress and inflammation, the recognition of both autoantigen and NKG2DL by NKG2D-expressing T cells causes the reduction of the T cell activating threshold (Figure 1). In this scenario, the expression of NKG2D ligands on stressed melanocytes is sensed by the immune system as an instruction for destruction (suicide by proxy). This initiation phase is followed by the perpetuation phase: once T cells have killed stressed melanocytes via engagement of both the TCR and NKG2D, these T cells are endowed with a transcriptional program that enables them to become long-lasting, with enhanced cytolytic properties as well as independence from immunological help. Evidence suggests that these NKG2D-engaged T cells are then responsible for the killing of healthy melanocytes in an NKG2D-independent mechanism, thus perpetuating the disease.

## Author Contributions

JG-P and LP-R prepared and wrote the manuscript. All authors contributed to the article and approved the submitted version.

## Conflict of Interest

The authors declare that the research was conducted in the absence of any commercial or financial relationships that could be construed as a potential conflict of interest.
