# Corrigendum: The Role of NKG2D in Vitiligo

**DOI:** 10.3389/fimmu.2021.714137

**Published:** 2021-06-09

**Authors:** Lourdes Plaza-Rojas, José A. Guevara-Patiño

**Affiliations:** ^1^ Department of Cancer Biology, Loyola University Chicago, Chicago, IL, United States; ^2^ Department of Surgery, Loyola University Chicago, Chicago, IL, United States

**Keywords:** vitiligo, NKG2D, T cells, horror autotoxicus, Hsp70, oxidative stress

There is an error in the article title. The correct title is The Role of NKG2D in Vitiligo.

The authors apologize for this error and state that this does not change the scientific conclusions of the article in any way. The original article has been updated.

